# Role of Autophagy and Apoptosis in Non-Small-Cell Lung Cancer

**DOI:** 10.3390/ijms18020367

**Published:** 2017-02-10

**Authors:** Guangbo Liu, Fen Pei, Fengqing Yang, Lingxiao Li, Amit Dipak Amin, Songnian Liu, J. Ross Buchan, William C. Cho

**Affiliations:** 1 Department of Molecular and Cellular Biology, University of Arizona, Tucson, AZ 85721, USA; peifen1990@gmail.com; 2 Department of Obstetrics and Gynecology, Dong’e No. 4 People’s Hospital, Liaocheng 252200, China; yfqldz@gmail.com; 3 Department of Medicine, Division of Hematology-Oncology, Sylvester Comprehensive Cancer Center, University of Miami Miller School of Medicine, Miami, FL 33136, USA; lingxiaoli@med.miami.edu (L.L.); amitdipakamin@med.miami.edu (A.D.A.); 4 Department of Chemical and Life Science Engineering, Virginia Commonwealth University, Richmond, VA 23284, USA; sliu6@vcu.edu; 5 Department of Clinical Oncology, Queen Elizabeth Hospital, Kowloon, Hong Kong, China

**Keywords:** non-small-cell lung cancer (NSCLC), apoptosis, autophagy, crosstalk, p53, mammalian target of rapamycin (mTOR), endoplasmic reticulum (ER) stress

## Abstract

Non-small-cell lung cancer (NSCLC) constitutes 85% of all lung cancers, and is the leading cause of cancer-related death worldwide. The poor prognosis and resistance to both radiation and chemotherapy warrant further investigation into the molecular mechanisms of NSCLC and the development of new, more efficacious therapeutics. The processes of autophagy and apoptosis, which induce degradation of proteins and organelles or cell death upon cellular stress, are crucial in the pathophysiology of NSCLC. The close interplay between autophagy and apoptosis through shared signaling pathways complicates our understanding of how NSCLC pathophysiology is regulated. The apoptotic effect of autophagy is controversial as both inhibitory and stimulatory effects have been reported in NSCLC. In addition, crosstalk of proteins regulating both autophagy and apoptosis exists. Here, we review the recent advances of the relationship between autophagy and apoptosis in NSCLC, aiming to provide few insights into the discovery of novel pathogenic factors and the development of new cancer therapeutics.

## 1. Introduction

Lung cancer is one of the leading causes of all cancer-related deaths. About 1.6 million people died from lung cancer in 2012, which accounts for 19% of all cancer death worldwide. In fact, lung cancer deaths alone exceed the sum of the next three most prevalent cancers death (colon, breast, and prostate) [1]. Lung cancer’s poor prognosis is partly due to late diagnosis that results from the lack of any obvious symptoms during early disease progression. In addition, multiple gene alterations including mutations, amplifications, deletions and fusions identified in NSCLC aggravate the abnormalities of signaling pathways and physiological activities. Histological classification sub-divides lung cancers into two subgroups, non-small-cell lung cancer (NSCLC, 85%) and small-cell lung cancer (SCLC, 15%), both of which are therapeutically treated differently. NSCLC is further divided into three main histological subtypes including squamous-cell carcinoma, adenocarcinoma, and large-cell carcinoma. Squamous-cell carcinoma accounts for 20%–30% and is usually identified in the center of the lung close to the air tube. Adenocarcinoma accounts for 40%–70% and is found at the outer area of the lung. Large-cell carcinoma accounts for 10%–15% of cases and can be found throughout the lung [2].

Autophagy and apoptosis play an important role during lung cancer progression. Macroautophagy (hereafter called autophagy) is a “self-eating” process, engulfing cytoplasmic proteins, complexes or organelles into the autophagosome (a cytoplasmic double membrane structure). The autophagosome is either incorporated into the late endosome, or is transported to, and fuses with, the lysosome to generate the autolysosome, where cargos are then released and degraded by acidic hydrolases. The degradation products generated, including nucleotides, amino acids, fatty acids and sugars, can then be transported back and recycled into general cell metabolism [3,4,5].

Apoptosis has been intensively investigated in the past three decades and is established as the major mechanism of development and programmed cell death. In 1972, Kerr et al. first used the term “apoptosis” to describe a distinct morphology of cell death [6]. Apoptosis is regulated by intracellular and/or extracellular signals and characterized by morphological changes of the cell targeted for death that include nuclear fragmentation and condensation, mitochondrial outer membrane permeabilization (MOMP), membrane blebbing, cell shrinkage and apoptotic body formation [7]. Studies in different model systems uncovered the essential role of apoptosis in normal development and homeostasis. During the development of the nematode *Caenorhabditis elegans*, for example, 131 cells undergo programmed cell death [8]. Programmed cell death is also required for eliminating interdigital webs in higher vertebrates during digit formation [9]. Moreover, apoptosis also plays a defensive role in immune reactions or eliminating damaged cells, serving as a quality control for homeostasis [10].

Autophagy can play a positive and negative role in promoting apoptosis in NSCLC. Generally, inhibition of autophagy limits the ability of cells to overcome stress and maintain homeostasis [11]. However, there are instances where autophagy promotes cell death, and these have been described elsewhere [12,13,14,15,16,17]. The interplay of autophagy and apoptosis in NSCLC is complicated. Indeed, autophagy functions as a double-edged sword in carcinogenesis. On the one hand, autophagy is always inhibited by different oncoproteins, such as AKT, PI3K, Bcl-1 and mutant p53, which may prevent excessive protein degradation in starved or stressed tumor cells. On the other hand, persistent activation of autophagy causes autophagic programmed cell death or apoptosis [18,19]. The processes of autophagy and apoptosis are discussed in detail below.

## 2. Autophagy and Apoptosis

### 2.1. The Mechanism of Autophagy

Autophagy starts with the formation of the isolation membrane/phagophore, which is often found at the contact sites between the endoplasmic reticulum and mitochondria [20]. The subsequent phases of the autophagic pathway include vesicle elongation, autophagosome maturation (sequestration of cargo), autophagosome-lysosome fusion and degradation [21]. Multiple molecular complexes perform the key processes of each phase of autophagy, including (but not limited to) ULK1 complex (ULK1, FIP200, ATG13L and ATG101), VPS34 complex (VPS15, VPS34, Beclin-1, ATG14 or UVRAG), ubiquitin-like conjugation system (ATG5, ATG12, ATG16L), and LC3 (type II light chain 3) conjugation system [22].

In physiological situations, autophagy is always occurring at a basal level, functioning as an intracellular quality control system to maintain homeostasis where any superfluous and/or damaged protein entities are removed [7]. Autophagic flux is always activated at, for example, transit cellular stress and nutrient starvation and is often visualized by the accumulation of the autophagosome [7,22,23,24,25]. The most commonly assessment to evaluate autophagic activity is the autophagosome accumulation, which can be reflect either accelerated autophagosome formation to increase the autophagic flux or slowed autophagosome clearance in the lysosome to decrease the autophagic flux [26]. For a detailed review of the apoptosis and autophagy pathway, please see other excellent recent reviews [5,7,10].

### 2.2. The Mechanism of Apoptosis

Apoptosis is the main and most well-studied form of programmed cell death. Caspases are ubiquitously expressed cysteine proteases that play a central role in apoptosis. In physiological scenarios, caspases are expressed as inactive forms with little protease activity [27,28]. Death-inducing stimuli lead to the cleavage at aspartic residues of caspases and removal of the N-terminal inhibitory domain, resulting in the demolition phase of apoptosis [28]. While caspases are predominantly associated with apoptosis, it is worth noting that some caspases are also involved in cytokine maturation and innate immunity [29].

Two core pathways of apoptosis include the extrinsic death receptor pathway and the intrinsic mitochondria pathway. The extrinsic pathway is initiated by the activation of multiple death receptors, such as Fas (CD95/Apo1), tumor necrosis factor receptors (TNFRs), and TNF-related apoptosis-inducing ligand receptors (TRAILRs). When death stimuli occur, the cytoplasmic domains of death receptors recruit death domain-containing adaptor proteins, such as FADD (Fas-associated protein with death domain) and TRADD (TNFR1-associated death domain), which then interact with pro-caspase-8 by the death effector domain (DED) to form the death-inducing signaling complex (DISC) to propagate the apoptotic signal. Pro-caspase-8 is activated in the DISC, which then directly proteolytically cleaves and activates caspase-3. Activated caspase-8 additionally triggers mitochondrial damage by the cleavage of the BH3-only protein BID to generate truncated BID (tBID) [30]. Activation of the intrinsic mitochondria pathway is marked by mitochondrial outer membrane permeabilization (MOMP), which in turn releases cytochrome c to the cytoplasm. Apaf-1 and pro-caspase-9 are recruited to cytoplasmic cytochrome c to generate the apoptosome with a 1:1:1 molar ratio [31]. The apoptosome activates the apoptosis effector protein caspases-3, -6 and -7, resulting in apoptosis.

The intrinsic apoptotic pathway is also tightly regulated by the Bcl-2 family proteins, which is comprised of pro-apoptotic members (such as Bax, Bak, Bad, Bcl-Xs, BID, Bik, Bim, HRK, Noxa and PUMA) and anti-apoptotic members (such as Bcl-2, Bcl-Xl, Bcl-W, Bfl-1 and MCL-1) [32]. Multiple cellular stresses, including DNA damage, energy starvation and hypoxia, dephosphorylates and cleaves these pro-apoptotic proteins, leading to their translocation to the mitochondria and consequent apoptosis. In addition, BH3-only proteins, such as Bim and BID, act through activation of pro-apoptotic Bax and Bak to induce apoptosis. Furthermore, indirect suppression of the anti-apoptotic proteins, such as Bad, Bik, Noxa, HRK and PUMA, in the mitochondria and endoplasmic reticulum can also cause apoptosis. Abnormalities in Bcl-2 family proteins are seen for example in NSCLC, where amplification of the anti-apoptotic Bcl-2 and MCL-1 proteins is associated with clinical drug resistance [33,34].

## 3. Crosstalk between Autophagy and Apoptosis

### 3.1. Linking Autophagy and Apoptosis

Autophagy and apoptosis occur when cells are under stress [7]. Normally, autophagy precedes apoptosis and maintains cell homeostasis. Apoptosis or other types of programmed cell death, is activated once stress is prolonged for a critical duration or exceeds the intensity threshold [7,11].

Interestingly, a substantial amount of evidence suggests that autophagy itself may be another cell death mechanism, termed autophagic cell death (ACD), highlighting a potential pro-death role for autophagy [7,13]. ACD, characterized by the absence of chromatin condensation, accumulated cytoplasmic vacuolization, LC3 lipidation and caspase-independent apoptosis, is usually suppressed by autophagy pathway inhibitors or knock out/down of core autophagy genes [7]. Notably, a few other forms of cell death are also termed “ACD”, which make it confusing and controversial [26]. For example, autophagy-associated cell death (massive cytoplasmic vacuolization is accompanied with cell death), autophagy-mediated cell death (autophagy triggers apoptosis pathway activation) and a distinct apoptotic mechanism (independent of apoptosis and necrosis) are also sometimes called “ACD” [26]. Furthermore, the autophagic roles of caspases and apoptotic functions of autophagy-related proteins (ATGs) create further overlap between both processes (see below) [35]. Autophagy may thus function as a guardian or executioner of apoptosis depending on the surrounding micro-environment, therapeutic intervention and the stage of carcinoma.

### 3.2. Beclin-1/Bcl-2 and FLIP (FADD-Like IL-1β-Converting Enzyme-Inhibitory Protein)

Various factors function in both apoptosis and autophagy. Beclin-1, for example, binds to Bcl-2 and inhibits both autophagy and apoptosis at basal levels. Nutrient starvation activates JNK1 (C-Jun N-terminal protein kinase 1), which phosphorylates the regulatory loop of Bcl-2 and then severs the interaction between Bcl-2 and Beclin-1. Isolated Beclin-1promotes autophagy by activation of core autophagic components, such as lipid kinase VPS34 and Beclin-1/VPS34/vps15 core complex. In addition, phosphorylated Bcl-2 interacts with Bax and maintains the integrity of mitochondrial membrane, promoting the anti-apoptotic functions [36]. However, extended starvation results in JNK1-mediated Bcl-2 hyper-phosphorylation, which leads to the dissociation with Bax and activation of caspase-3-mediated apoptosis [37]. In addition, cardiac glycoside ouabain, a Na/K-ATPase inhibitor, induces Bcl-2 reduction due to JNK1 activation, which disrupts Bcl-2/Beclin-1 interaction and results in caspase-independent autophagic cell death in NSCLC cells [38].

FLIP (FADD-like IL-1β-converting enzyme-inhibitory protein) is an anti-apoptotic protein involved in death receptor-mediated extrinsic pathway [39]. In addition, FLIP inhibits LC3 lipidation by competitive interaction with ATG3, which in turn blocks autophagy. Conversely, induction of autophagy suppresses FLIP and ATG3 interaction [40].

### 3.3. The Role of Autophagy-Related Proteins (ATGs) in Apoptosis and Autophagy

Autophagy is a highly conserved process in organisms, from yeast to humans, which relies on the function of a core set of ATGs [41]. However, apoptotic roles for several ATGs have also been recently described. For example, Atg4 is a cysteine protease identified in yeast that is involved in autophagosome formation. ATG4D is its human ortholog which is cleaved by caspase-3 during apoptosis. The C-terminal of cleaved ATG4D contains a putative BH3 domain, which is recruited to the mitochondria to stimulate apoptosis [42]. In addition, ATG12-ATG3 is required for the formation of the autophagosome. The complex can also localize at the outer membrane of mitochondria to stimulate intrinsic apoptosis [43]. Furthermore, covalent conjugation of ATG5 and ATG12 is involved in an ubiquitylation-like process that is essential for autophagy induction [44]. Interestingly, ATG5 and ATG12 can induce apoptosis upon stress. ATG5 overexpression promotes tumor cell sensitivity to chemotherapy, whereas inhibition of ATG5 increases resistance to chemotherapy [35]. In addition, apoptosis induction inhibits the autophagic role of ATG5 and promotes ATG5 cleavage by calpains. The amino-terminal cleaved product of ATG5 translocates from the cytoplasm to mitochondria, resulting in its association with Bcl-Xl and the release of cytochrome c to the cytoplasm, leading to apoptosis [45]. Following apoptotic stimuli, non-conjugated ATG12 binds to MCL-1 and Bcl-2, thereby inhibiting their anti-apoptotic functions and augmenting mitochondrial-mediated intrinsic apoptosis [46].

### 3.4. The Role of Caspases in Autophagy and Apoptosis

Recently, it has been shown that caspases participate in the regulation of autophagy as well [35,47]. Activated caspases result in autophagy inhibition by breaking down autophagy-associated proteins, such as Beclin-1, ATG5 and p62. Caspase-8, a well-known essential apoptotic protein, regulates autophagy by cleavage of ATG3, leading to the pro-autophagic inhibition. Prevention of caspase-8 cleavage and activation induces hyperactive autophagy in T cells [48]. Caspase-9, another apoptosis protein associated with apoptosome formation and intrinsic apoptosis pathway, induces autophagy by increasing LC3 lipidation via an interaction with ATG7 [35,41]. Conversely, the combination of caspase-9 and ATG7 inhibits translocation of caspase-9 to the apoptosome, hence preventing apoptosis [35,41]. In addition, the predominant apoptotic protein caspase-3 also shows autophagic activity. Caspase-3 cleaves Beclin-1 at TDVD^133^ and DQLD^149^, yielding fragments lacking pro-autophagic capacity. It is worth noting that the C-terminal of Beclin-1 localizes at the mitochondria and augments apoptosis [49]. Therefore, activated caspases not only perform apoptosis, but also hinder or reinforce autophagy by their interaction with and cleavage of autophagy-related proteins. Immunoprecipitation of key apoptotic proteins, such as caspase-8, caspase-3 and caspase-9 upon stimulation—including nutrient starvation, hypoxia or chemical treatment—will help to further our understanding of the crosstalk between autophagy and apoptosis.

## 4. The Abnormality of Genome in Non-Small-Cell Lung Cancer (NSCLC)

Nowadays, the ability to sequence the genomes of NSCLC patients has given a greater insight into the disease through the identification of aberrant genes, leading to new biomarkers and therapeutic targets [2,50]. Table 1 summaries known mutant genes in both adenocarcinoma and squamous-cell carcinoma. The most commonly mutated gene is *p53*, seen in 45%–70% of adenocarcinomas and 60%–80% of squamous-cell carcinoma. Other aberrant genes, such as *EGFR*, *KRAS*, *LKB1* and *PTEN*, are closely associated with the mTOR regulation network. Hence, we will discuss p53 in this section and mTOR pathway in Section 5.

### 4.1. p53 Function

**Figure 1:**
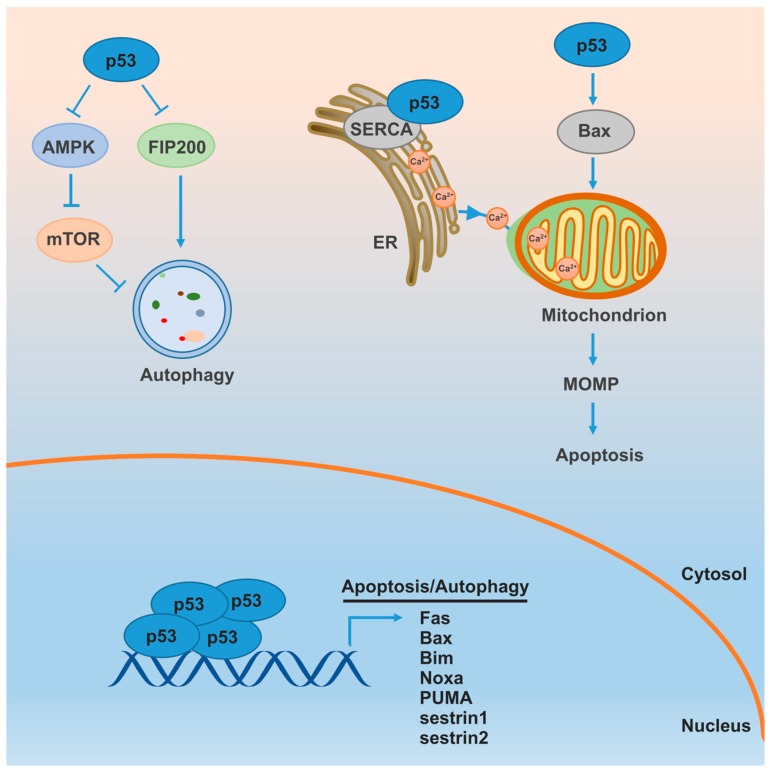
p53 promotes apoptosis and inhibits autophagy. Nuclear p53 induces autophagy and the transcription of multiple apoptotic genes that function in both extrinsic and intrinsic apoptosis pathway. Cytoplasmic pools of p53 directly or indirectly trigger MOMP and lead to apoptosis. In addition, cytoplasmic p53 inhibits autophagy by FIP200 or the AMPK/mTOR pathway.

p53 is normally located in the cytoplasm and translocates to the nucleus following the direct or indirect phosphorylation by a variety of kinases upon cellular stress [51]. Phosphorylated nuclear p53 forms a tetramer and functions as a transcription factor that promotes the expression of a variety of pro-apoptotic proteins mediating both death receptor and mitochondrial pathways, such as Fas, Bax, Bim, Noxa and PUMA (Figure 1) [52]. In addition, MOMP can also be modified by cytoplasmic monomeric p53 via regulating MOMP-governing Bcl-2 family proteins. Here, p53 translocates to the mitochondrial surface and directly binds to Bcl-2 family proteins, leading to apoptosis [53,54]. The intrinsic pathway plays the major role in p53-mediated apoptosis, although p53 can also regulate the extrinsic pathway. Besides controlling the Bcl-2 family, p53 also promotes Apaf-1 and caspase-6 expression, resulting in apoptosis [55]. Recently, the apoptotic effect of cytoplasmic p53 has been associated with the endoplasmic reticulum (ER), which is a critical organelle modulating autophagy and apoptosis, discussed further in Section 6. Wild-type p53 translocates to the ER and mitochondria-associated ER membranes during the stimulation, and directly interacts with the sarco/ER Ca^2+^-ATPase (SERCA) pump to increase Ca^2+^ load. More Ca^2+^ accordingly transfers to the mitochondria, leading to mitochondrial morphology alterations and apoptosis due to Ca^2+^ overload [56,57].

p53 can also regulate autophagy. Nuclear p53 promotes autophagy whereas cytoplasmic p53 inhibits it. Following stress, p53 translocates to the nucleus and promotes sestrin1 and sestrin2 transcription and expression, which can act through AMPK-TSC2-mTOR pathway to stimulate autophagy [58]. Cytosolic p53 interacts with the autophagy component FIP200 and competitively inactivates autophagy. The anti-autophagic role of p53 can also be modulated by inhibition of AMP-dependent kinase (positive regulator of autophagy) and activation of mTORC1 (negative regulator of autophagy) [59]. Cell lines and mouse model show that blocking p53 expression with pifithrin-α induces autophagy [60]. p53 knockout cells display enhanced autophagy compared to its wild-type counterpart as well. Conversely, inhibition of p53 degradation prevents the induction of autophagy. Furthermore, p53 inhibition-mediated autophagy protects cells from apoptosis upon hypoxia or nutrient starvation, indicating an anti-apoptotic role of autophagy [61].

### 4.2. p53 Mutation

The frequent loss of heterozygosity (LOH) of p53 on chromosome 17p13 suggests p53 is likely involved in the pathogenesis of NSCLC [62]. Hence, understanding the p53 network may ultimately allow scientists and clinicians to develop novel drugs and therapeutics. p53 was identified in 1979 and functioned as the tumor-suppressor gene although it was initially considered as the oncogene for the first ten years [63,64,65,66]. Wild-type p53 not only limits its transforming ability, but also inhibits transformation mediated by the classic oncogenic protein, KRAS [62]. Mutant p53 is commonly observed in tumors with poor prognosis, increased malignancy and resistance to treatment [67,68,69,70,71,72,73]. Experiments in both rodent and human cell lines show that mutant p53 can transform primary cultured cells through loss of its tumor-suppressive functions [74]. Loss of function (LOF) with wild-type p53 and gain of function (GOF) with mutant p53 may also aid in tumorigenesis. Abolishing mutant p53’s oncogenic properties or conversion of mutant p53 to the wild-type form is a potential therapeutic approach [71,75].

Different mutant p53 alleles can localize to the nucleus and/or cytoplasm, which are unable or able to inhibit autophagy depending on cell context and environment. In addition, some p53 mutations, including L22Q and W23S, retain its pro-apoptotic properties, whereas other mutations, including L194F and R280K, lose this, which may augment tumorigenesis and disease progression [51,56]. Similarly, mutant (such as R172H and R270H) and p53 deletions when combined with mutant KRAS inhibit apoptosis and promote tumor metastasis in genetic lung cancer mouse models and patients [76,77]. In addition, the NF-κB signaling pathway, which is associated with inflammation, and apoptosis, is also stimulated by mutant p53 with KRAS expression in murine lung cancer models [72,78]. Finally, mutant p53 can promote mitochondrial activity and antioxidant capacity, correlating with poor clinical outcomes [79,80].

### 4.3. p53 and Smoking

Smoking is the number one risk factor of lung cancer incidence. In the United States and European Union, about 90% of males and 75% of females diagnosed with lung cancer are smokers [80,81,82]. Genetic research shows tobacco-associated cancer patients have a higher frequency of p53 mutations compared with the tobacco-free patients (26%–71% compared to 8%–47%) [83]. Furthermore, mutant p53 is highly correlated with aggressive disease and shorter survival, indicating mutant p53 loses its tumor-suppressor role [84,85,86,87].

Genetic screening has revealed that the transversion of G:C to T:A is a characteristic hotspot in tobacco-associated lung cancer [87,88,89]. These mutations are commonly found in tobacco-related lung cancer at codons 157, 158 and 273, but are significantly rarer in never-smokers [87,90]. Of note, the forms of mutant p53 are different between smokers and never-smokers. Transversions and deletions account for 80% of p53 mutations in smoking women, whereas p53 transitions account for 80% in tobacco-free adenocarcinoma women [91]. Notably, while p53 mutations are often found in smokers, EGFR mutations are more frequent in females and never-smokers with adenocarcinoma. Around 15% of males and 50% of females with lung cancer are never-smokers [71,92]. Accordingly, different therapeutics must be developed that are tailored to the different p53 mutant subtypes. In addition, mutant epidermal growth factor receptor (EGFR) makes up 10%–40% in adenocarcinoma. EGFR abnormalities act through the PI3K/AKT/mTOR (phosphoinositide 3-kinase/protein kinase B/mammalian target of rapamycin) and Raf/MEK/ERK (raf/mitogen-activated protein kinase kinase/extracellular signal-regulated kinases) pathways to drive oncogenesis and are discussed below.

### 4.4. Compounds Targeting p53 in NSCLC

Exploring new compounds that target mutant p53 and restore its wild-type function are potential therapeutic strategies for cancers, especially NSCLC, due to the high mutation rate of p53 [55,93,94]. Several compounds have been shown such therapeutic potential. Nutlin, for example, is a compound capable of increasing wild-type p53’s anti-tumor activity by blocking the interaction between p53 and MDM2 in vivo (E3 ubiquitin ligase of p53) [95]. Mammalian cell lines and mouse xenograft models show that PRIMA (p53 reactivation and induction of massive apoptosis), identified over 10 years ago, can bind to and convert mutant p53 to its wild-type structure, leading to growth inhibition and apoptosis [96,97]. RETRA (reactivation of transcriptional reporter activity) is another compound which inhibits mutant p53 activity by releasing p73 (p53 family protein with a high level of sequence similarity) from the p53 complex and activating target proteins associated with growth inhibition and apoptosis induction [98]. Hence, developing and discovering new molecules targeting abnormal p53 or promoting the pro-apoptotic role of wild-type p53 can aid clinical cancer therapy. Most p53 mutations occur at the DNA-binding domain (DBD), resulting in loss of DNA binding activity and transactivation function. Hence, restoring and stabilizing the DBD structure is also a promising strategy to restore wild-type p53’s tumor-suppressor function.

## 5. Mammalian Target of Rapamycin (mTOR) Pathway

**Figure 2:**
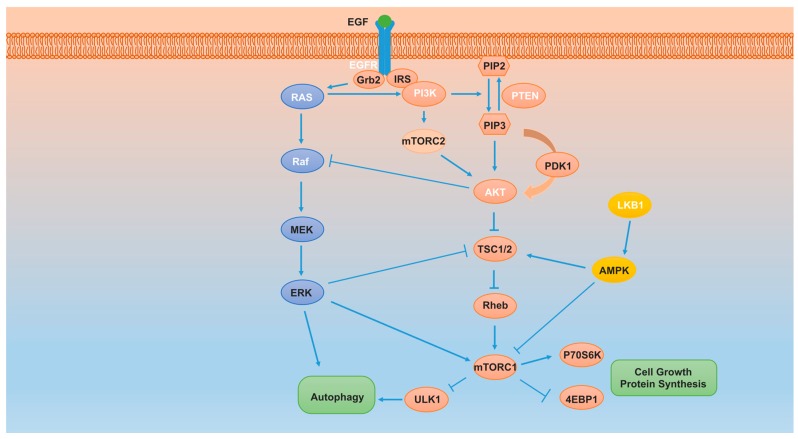
Overview of mammalian target of rapamycin (mTOR) pathway. The crosstalk of different pathways including (PI3K)/ (PI3K/AKT)/mTOR (phosphoinositide-3-kinase-protein/kinase B/mTOR), LKB1/AMPK/mTOR (serine/threonine kinase 11/ AMP-activated protein kinase/mTOR) and Raf/MEK/mTOR (rapidly accelerated fibrosarcoma/ mitogen-activated protein kinase kinase/mTOR) and their regulation of autophagy. Aberrant genes associated with NSCLC are marked in white. Hexagons and ovals are representative of lipids and proteins, respectively. Arrows indicate stimulation and T-bars indicate inhibition. PtdIns(3,4,5)P_3_ (PIP3) can activate AKT directly or recruit 3-phosphoinositide-dependent protein kinase-1 (PDK1) to phosphorylate AKT.

The mammalian target of rapamycin (mTOR) signal transduction pathway is involved in a variety of cellular functions upon either intracellular or extracellular stimulation (Figure 2). Numerous alterations in genes such as *KRAS*, *EGFR*, *LKB1*, *PTEN*, *PIK3CA* (encoding the p110α catalytic subunit of PI3K), as well as *AKT1* mutations, *EGFR* and *PIK3CA* amplification, and *PTEN* deletion, have been described in NSCLC, which lead to uncontrolled mTOR pathway signaling. Dysregulation of the mTOR pathway is more common in squamous lung carcinoma than adenocarcinoma (Table 1) [99,100]. Furthermore, patients carrying mutant EGFR always exhibit aberrant PI3K/AKT/mTOR activation, which causes resistance to EGFR-tyrosine kinase inhibitor (EGFR-TKI) treatment in clinic [99].

### 5.1. mTOR Function

mTOR is a conserved serine/threonine kinase associated with multiple physiological functions, such as cell cycle regulation, proliferation, differentiation, motility and invasion. mTORC1 (composed of mTOR, Raptor, Deptor, mLST8 and PRAS40) and mTORC2 (made up of mTOR, Rictor, Deptor, mLST8, Sin1, and PRA5/Protor-1) are two signaling complexes involved in the mTOR pathway. Raptor and Rictor are scaffold proteins involved in mTORC1 and mTORC2 assembly, respectively. mTORC2 mediates AKT phosphorylation at Ser473 to activate mTORC1. Alternatively, activated mTORC1 sensitizes ribosomal protein S6 kinase (S6K), which in turn stimulates the mTORC2 complex. In addition, mTORC1 is energy and stress sensitive and robustly inhibited by rapamycin whereas mTORC2’s insensitivity to rapamycin and nutrients due to the existence of Rictor [101]. However, prolonged and chronic rapamycin treatment will eventually suppress mTORC2 activity [102].

Numerous studies show that an important function of mTORC1 is the negative regulator of autophagy [99,103]. In normal and nutrient-rich conditions, mTORC1 phosphorylates UNC-51-like kinase 1(ULK1) to suppress its pro-autophagic role. The pro-autophagic ATG13, which positively regulates ULK1, can be directly phosphorylated and inhibited by mTORC1 as well. In glucose or amino acid starved conditions, AMPK can directly regulate the ULK1 or VPS34-Beclin-1-ATG14 complexes to promote autophagy or indirectly inhibit mTOC1 activity and then promote ULK1-mediated phosphorylation of FIP200 and ATG13. Furthermore, ULK1 can also mediate autophagy through phosphorylation of Beclin-1 to form the VPS34-Beclin-1-ATG14 complex to activate autophagy [99,104,105].

mTOR is innately considered as a pro-survival factor and acts as an inhibitor of apoptosis. Following stress, mTOR depletion inhibits cell growth and proliferation and increases autophagy and apoptosis. In contrast, cytoplasmic p53 inactivates mTOR signaling and suppresses autophagy. The induction of autophagy is normally a self-protective process that acts to counteract apoptosis. Persistent and high levels of autophagy, however, oppose this counteraction, and instead act synergistically with apoptosis to cause cell death. Since a variety of intracellular and extracellular signals, energy status, different stresses and crosstalk with other signaling pathways affect mTOR activity, combining mTOR inhibitors with pro-apoptotic or anti-autophagic molecules may be highly efficacious.

### 5.2. Effect of Phosphoinositide-3-Kinase-Protein/Kinase B (PI3K/AKT) Signaling on the mTOR Pathway

The phosphoinositide-3-kinase-protein (PI3K)/kinase B (AKT) signaling pathway was identified in the 1980s and plays an important role in many different physiological activities, such as protein synthesis, metabolism, cell cycle regulation, proliferation and apoptosis [106]. A variety of upstream signals, such as insulin-like growth factor-1 (IGF-1), human epidermal growth factor receptor (EGFR) and vascular endothelial growth factor receptors (VEGFRs) act through the PI3K-AKT pathway to regulate mTOR activity [107]. During this process, class 1A PI3Ks (PI3Kα, β, and δ) are stimulated by the activated receptors and interact with the intracellular domain of receptors by adapter molecules, such as insulin receptor substrate (IRS) and growth factor receptor-bound protein 2 (Grb2). PtdIns(4,5)P_2_ (PIP2), a membrane phospholipid component, is converted to PtdIns(3,4,5)P_3_ (PIP3) by PI3K-mediated phosphatidylinositols (PtdIns) modification at the 3-position. Conversely, 3-phosphatase PTEN dephosphorylates PIP3 and regenerates PIP2. In addition, SHP1/2 help to generate PtdIns(3,4)P_2_ by dephosphorylating at the 5-position [99,108,109,110].

PIP3 is an important intracellular second messenger and activates targeting proteins, such as AKT and PDK1 (3-phosphoinositide-dependent protein kinase-1, a serine/threonine kinase contains PH domain). Three isoforms of AKT have been identified, AKT1 (which is ubiquitously expressed), AKT2 and AKT3 (which are highly expressed in insulin responsive tissues or the brain and testis, respectively) [111]. AKT is a serine/threonine kinase composed of an amino-terminal pleckstrin homology (PH) domain, central catalytic domain and carboxyl-terminal regulatory domain. Newly synthesized PIP3 is anchored on the inner leaflet of the plasma membrane and directly interacts with the PH-domain of AKT. In addition, PDK1 is also recruited on the membrane and phosphorylates AKT at Thr308, which stimulates AKT activity [112]. Following the stimulation, PRAS40 and TSC2 are directly phosphorylated and inactivated, which in turn activate mTORC1 and promote protein synthesis and proliferation via the phosphorylation of ribosomal protein S6 kinase (S6K) and eukaryotic initiation factor 4E (eIF4E)-binding protein 1 (4EBP1) [109,113]. In addition, the maximal activity of AKT can be achieved by mTOR or PDK2-mediated phosphorylation at AKT Ser473 [114,115]. In contrast, protein phosphatase 2 (PP2A) and PH Domain Containing Leucine Rich Repeat Protein Phosphatase 1/2 (PHLPP1/2) antagonize AKT phosphorylation at Thr308 or Ser473, respectively, preventing its activation [116,117].

### 5.3. Effect of Liver Kinase B1/AMP-Activated Protein Kinase (LKB1/AMPK) Signaling on the mTOR Pathway

mTOR can also be regulated in an AKT-independent manner, for example through the LKB1/AMPK pathway [118,119]. The mutation or deletion of LKB1, a serine/threonine master kinase, has been found in NSCLC, which may cause loss of its tumor-suppressor function and promote tumor growth via the LKB1/AMPK/mTOR pathway [100,120,121]. One of the direct substrates of LKB1 is AMPK (AMP-activated protein kinase), which is an intracellular energy sensor and cell homeostasis monitor [118,122]. As a heterotrimeric serine/threonine kinase, AMPK contains a catalytic α subunit and two, β and γ, regulatory subunits. Following nutrient deprivation, increased intracellular AMP and decreased ATP induce AMPK activation. LKB1 is involved in this process by phosphorylation at Thr172 of α activating loop [119]. Activated AMPK controls several processes, such as p53 phosphorylation [123], sirtuin1 activity [124], fatty acid, cholesterol synthesis [125] and mTOR pathway regulation. AMPK can directly phosphorylate tuberous sclerosis complex 2 (TSC2) or Raptor to inhibit mTOC1 activity [118,126,127,128]. During this process, the small GTPase Rheb (Ras homolog enriched in brain) is the targeting protein that propagates the mTORC1 inhibition signal emitted by TSC2.

### 5.4. Effect of Raf/MEK/ERK Signaling on the mTOR Pathway

The Raf/MEK/ERK (rapidly accelerated fibrosarcoma/mitogen-activated protein kinase kinase/extracellular signal-regulated kinase) mitogen-activated protein kinase cascade is also associated with mTOR regulation, controlling cell survival, differentiation and apoptosis. Raf and RAS mutations cause constitutive activation of Raf/MEK/ERK leading to tumorigenesis [129]. RAS is a small GTPase and functions as the cyclic switch between a GDP-bound inactive and GTP-bound active form. Multiple upstream factors, such as receptor tyrosine kinases, growth factors, heterotrimeric G-proteins, integrin, serpentine receptors and cytokine receptors can activate RAS. In addition, mutations in RAS slow the transition from GTP to GDP, leading to the constantly high activity of RAS [129]. Stimulated RAS can either evoke PI3K/AKT/mTOR pathway output or recruit and elicit Raf (MAPKKK) expression at the cell membrane. Raf then phosphorylates and activates MEK (MAPKK, intermediate kinase), which subsequently phosphorylates ERK (MAPK). Activated ERK then translocates to the nucleus and induces target gene transcription and expression, leading to cell survival, proliferation, and autophagy [30,129]. In addition, the Raf/MEK/ERK pathway can also activate mTORC1 activity by regulating PI3K, TSC2 and mTORC1. Conversely, AKT can phosphorylate the N-terminus of Raf, inactivating the Raf/MEK/ERK axis. Furthermore, the ERK and mTORC1 pathways can regulate the same downstream targeting proteins, such as Bad, GSK3 and YB1 [129]. This crosstalk and interaction indicate combining inhibitors against both pathways may result in an improved therapeutic outcome.

### 5.5. Molecules Targeting mTOR in NSCLC

There is great potential in inhibiting the mTOR pathway for cancer therapy [99]. Rapamycin (sirolimus), isolated in 1975 and named after the island—Rapa Nui—where it was discovered [108], strongly inhibits TOR kinase activity. Moreover, its immunosuppressive property has promoted it to application in clinical trials in the hopes that it will help counteractcancer and prevent organ transplant rejection [108]. Rapamycin can activate p53-independent mitochondrial-mediated apoptosis in NSCLC cells, highlighting its efficacy in this subtype of lung cancer [130]. The A549 NSCLC mouse model also shows decreased tumor growth and apoptosis upon rapamycin treatment [131].

The combination of rapamycin with other anti-cancer drugs can further increase its sensitivity and efficacy when compared to single-drug treatment. The Bcl-2 inhibitor ABT-737, for example, when combined with rapamycin, promotes apoptosis and autophagy, thereby improving radiation therapy both in vitro and in xenograft lung cancer models [132]. Rapamycin also inhibits thymidylate synthase expression and, when combined with pemetrexed (a widely used drug for NSCLC treatment), both drugs inhibit NSCLC cancer cell growth in both in vitro and in vivo models [133]. Recently, the combination of rapamycin with lipophilic bisphosphonates has been shown to be highly therapeutic in KRAS-mutant lung cancers both in vitro and in vivo [134]. Lipophilic bisphosphonates block prenylation of KRAS due to inhibition of both farnesyl and geranylgeranyldiphosphate synthases, which are required for cell growth. Bisphosphonate activates autophagy initiation, although it is ultimately unsuccessful in being completed by p62 accumulation (p62, a specific target of autophagic flux). The combination of bisphosphonate with rapamycin facilitates autophagy and significantly inhibits KRAS mutation-mediated tumor growth [134]. In addition, The EGFR tyrosine kinase inhibitor (TKI) erlotinib is regularly used in treating NSCLC patients. Using p53 null H1299 and wild-type p53 A549 cell-line models, erlotinib displays less sensitivity in H1299 cells, indicating growth inhibition is partly due to p53. Combining erlotinib with rapamycin overcomes the resistance to EGFR TKIs caused by the lack of p53 in vitro. Decreased cell proliferation upon dual treatment is accompanied with mitochondrial hyperpolarization and autophagy induction [135]. In addition, it has been shown that autophagy is not fully activated in the highly EGFR-TKI-resistant cells; therefore, co-treatment with rapamycin could enhance autophagy and restore the sensitivity to EGFR-TKIs [136].

Besides rapamycin, targeting proteins upstream of mTOR have also been studied in NSCLC, such as AZD8055 (PI3K inhibitor), NVP-BEZ235 (PI3K and mTORC1 inhibitor), Perifosine (AKT inhibitor) and GSK-690693 (AKT inhibitor). AZD8055 is an ATP-competitive mTOR inhibitor with excellent selectivity against class I PI3K isoforms and other PI3K-like kinase members [137,138]. AZD8055 accelerates autophagosome formation and autophagy activation versus rapamycin. It also shows more robust growth inhibition and tumor regression in NSCLC xenograft models [137]. In addition, AZD8055 treatment also induces autophagy and cell death in leukemia and breast cancer [139,140].

NVP-BEZ235, another PI3K inhibitor, recently entered clinical trials for breast cancer, renal cell carcinoma and prostate cancer. Inhibition of PI3K/mTOR by NVP-BEZ235 inhibits the expression of the anti-apoptotic MCL-1 and promotes activation of the pro-apoptotic Bim [141]. Accumulating evidence suggests that combining NVP-BEZ235 with other drugs targeting pro-proliferation pathways promote its cytostatic activity causing cytotoxicity and apoptosis in NSCLC. For example, its combination with the MEK inhibitor (AZD6244) induces expression of the pro-apoptotic Bim which, accordingly, enhances apoptosis. NVP-BEZ235 plus targeted STAT3 inhibition significantly sensitizes NSCLC cells to apoptosis, which is achieved via increased CHOP (C/EBP homologous protein), which is the ER stress-targeting protein [142]. In addition, NVP-BEZ235 mediates autophagy by counteracting its pro-apoptotic role. Combining it with the autophagic inhibitor chloroquine promotes stronger growth inhibition of human lung cancer cells [143].

Perifosine (KRX-0401) is an AKT and MAPK inhibitor that induces apoptosis in NSCLC cells by downregulation of AKT and increased output of the TRAIL receptors’ mediated extracellular apoptotic pathway [144]. Perifosine can also inhibit mTOR/Raptor and mTOR/Rictor assembly to reduce mTOR, Raptor, Rictor and 4EBP1 levels by tumor-suppressor FBW7, which is the ubiquitin ligase complex component. Furthermore, perifosine induces autophagy, evident by the increase of LC3. Interestingly, combining perifosine with lysosomal inhibitors enhances its anti-cancer effect [145,146], similar to the anti-apoptotic role of autophagy when treated with NVP-BEZ235. In addition, GSK-690693, an AKT kinase inhibitor, induces apoptosis in NSCLC cells accompanied by cytoprotective autophagy as well [147]. Moreover, multiple clinical trials of NSCLC, which target the mTOR pathway, are also in progress. For example, ABBV-221 (target EGFR), Navitoclax (target Bcl-2, Bcl-x and Bcl-w), Selumetinib (target Raf/MEK/ERK) and INK128 (target TORC1/2) are in phase 1 NSCLC clinical trials [148].

## 6. Endoplasmic Reticulum (ER) Stress

### 6.1. The Mechanism of ER Stress

The endoplasmic reticulum (ER) is the principal organelle required for protein folding, translocation modification, calcium homeostasis and lipid biosynthesis [149]. A variety of cellular stresses, such as oxidants, glucose deprivation, abnormal calcium regulation (e.g., ER-cytoplasm calcium release), viral infection, hypoxia, high-fat diet and biochemical reagents, disturb cell homeostasis and result in unfolded or misfolded protein accumulation in the ER, leading to the activation of the unfolded protein response (UPR), causing cells to rapidly adapt by speeding up the process of protein folding and limiting new protein synthesis [149,150,151]. Persistent and high levels of cellular stress, however, induce apoptosis, degrading the damaged cells. Both intrinsic and extrinsic apoptotic pathways and autophagy are elicited by ER stress.

**Figure 3:**
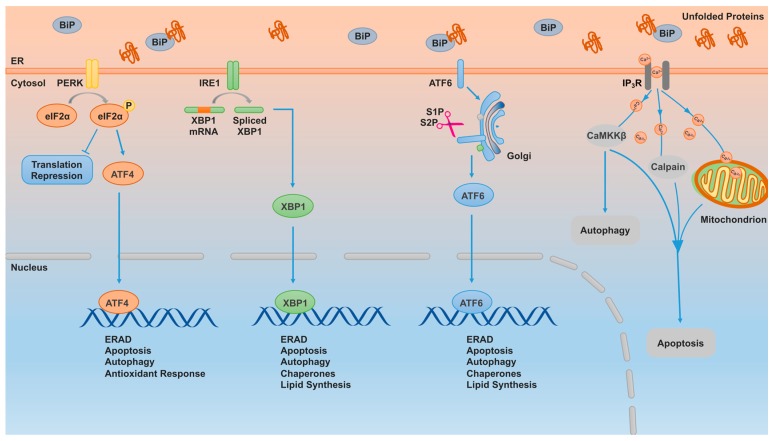
ER stress mediates apoptosis and autophagy.

There are three conserved ER transmembrane branches involved in signaling ER stress, including inositol-requiring enzyme 1α (IRE1α), activating transcription factor 6 (ATF6) and pancreatic ER kinase-like ER kinase (PERK) (Figure 3) [150]. The binding immunoglobulin protein (BiP) (also called GRP78 or Kar2p) is a chaperone protein that binds with the luminal domains of the transducers IRE1α, ATF6, and PERK under normal conditions. In ER-stressed cells, BiP disassociates from these transducers and binds with the unfolded or misfolded proteins that accumulate in the lumen to assist in protein refolding and to restore cell homeostasis. Consequently, BiP-disassociated IRE1α is phosphorylated causing homo-dimerization and subsequent activation of its kinase and RNase activities that cytoplasmically splice XBP1 mRNA, thereby activating it. Newly synthesized XBP1 then translocates to the nucleus and induces gene expression of factors associated with chaperones and ER-associated protein degradation (ERAD). Similarly, released ATF6 moves to the Golgi compartment and is cleaved by the proteases sphingosine-1-phosphate (S1P) and sphingosine-2-phosphate (S2P) yielding the activated form of ATF6 that regulates target gene transcription and expression. Finally, following disassociation with BiP, PERK forms homodimers, is auto-phosphorylated and subsequently phosphorylates eIF2α, inhibiting the initiation of translation for most mRNAs, but activating ATF4 mRNA translation via a uORF-dependent mechanism [152]. ATF4 then translocates to the nucleus and functions as a transcription factor to stimulate anti-oxidative and apoptotic protein expression. The activation of ER stress can also elicit extrinsic apoptosis pathway activation, for example, through the induction of the death receptor 5 (DR5). Interestingly, DR5 is involved in the control of ER stress-mediated apoptosis as well [39,153,154,155].

The ER is also involved in intracellular Ca^2+^ homeostasis. Following ER stress, calcium is released from the lumen to the cytosol through Ca^2+^ channels, such as Inositol trisphosphate receptor (IP3R). Increased cytoplasmic calcium promotes calpain expression, which is a ubiquitously expressed cysteine protease. Calpain then acts through the caspase-4/caspase-9/caspase-3 axis to induce apoptosis. In addition, cytoplasmic calcium increases the mitochondrial calcium concentration and causes abnormal mitochondrial membrane potential. Furthermore, the released calcium elicits CaMKKβ (Calcium/calmodulin-dependent protein kinase) expression, which activates the AMPK/mTOR pathway resulting in autophagy and apoptosis [156].

### 6.2. Autophagy, Apoptosis and ER Stress in NSCLC

Several studies show that ER stress is a crucial regulator of apoptosis and autophagy. For example, curcumin, a phenolic compound isolated from the plant *Curcuma longa* and one of the most promising anti-cancer agents, induces apoptosis and cell cycle arrest through ER stress in A549 and H460 cells [157,158,159]. In addition, curcumin treatment elicits autophagy in lung adenocarcinoma A549 cells which has a pro-death effect alongside apoptosis [160]. Two ER stress proteins, CHOP and BiP, are stimulated upon curcumin treatment. Curcumin also stimulates ER calcium release into the cytoplasm, resulting in changes in mitochondrial membrane potential, which releases cytochrome c and endonuclease G (endo G), which could promote caspase cascade activation or nuclear DNA degradation. Curcumin can also directly activate the Fas-mediated extracellular apoptotic pathway to induce apoptosis [157,158]. In addition, CLEFMA is a synthetic curcumin mimic and induces autophagy in H441 cells, as well [161].

Salinomycin, an extracted potassium ionophore from *Streptomyces aibus*, promotes the ATF4-DDIT3/CHOP-TRIB3 axis and suppresses AKT1 and mTOR activity, activating autophagy and apoptosis in NSCLC cells. Inhibition of autophagy by knockdown of ATG5 or ATG7 enhances salinomycin-mediated apoptosis, indicating their autophagic cytoprotective roles [162]. Similarly, H1, a bromized derivative of tetrandrine, induces ER-stress-mediated DR5 expression and apoptosis in NSCLC cells. Simultaneously, autophagy is stimulated and functions as anti-apoptotic during this process [163]. Cytoprotective autophagy in NSCLC cells has also been described upon treatment with other molecules, such as cucurbitacin E and glycerrhetinic acid [164,165].

In contrast, methyl jasmonate, a botanical hormone, causes ER stress activation and pro-apoptotic autophagy in NSCLC cells. Cryptotanshinone or isocryptotanshinone treatment in NSCLC cells also induce pro-apoptotic autophagy [13]. It is important to note that the induction of autophagy may neither be pro- nor anti-apoptotic. Licochalcone A, a flavonoid extracted from Chinese medicinal herb *Glycyrrhiza uralensis* Fisch, induces ER stress-mediated apoptosis and autophagy; however, the autophagic effect neither promotes nor inhibits apoptosis in NSCLC cells [166]. Together, the anti-, pro- or non-apoptotic role of autophagy is dependent upon different molecules. These mechanisms are still poorly understood and warrant further investigation.

## 7. Therapeutic Approaches in NSCLC

NSCLC is the most common cancer worldwide and is characterized by high resistance to chemo- and radiation therapy, resulting in a high mortality rate. The success of immune checkpoint blockade in the clinic has encouraged the rapid development of cancer immunotherapeutics [167,168]. Here we will briefly talk about promising immunotherapy approaches, and briefly highlight promising Chinese medicinal approaches.

### 7.1. Immunotherapy in NSCLC

Recently, immunotherapy that stimulates the immune response and inhibits tumor growth has attracted significant attention, and opened another door in the fight against cancer, including NSCLC. In this process, tumor cells are recognized by the immune system, leading to not only tumor removal, but also restoration of the surveillance ability of immune system, which is typically suppressed during cancer development. Immune checkpoints are regulators that play an important role in preventing autoimmunity, detrimental inflammation and maintaining self-tolerance. In the carcinogenesis process, immune checkpoints are co-opted by tumors, resulting in immune tolerance and malignant development [169]. Hence, blocking immune checkpoints in cancer cells provides new immunotherapeutic targets.

PD-1 (programmed death receptor 1) and CTLA-4 (cytotoxic T-lymphocyte antigen 4) are two well-characterized checkpoints in NSCLC clinical trials. Normally, PD-1 inhibition is found at the site of the tumor, whereas CTLA-4 occurs primarily in the lymphoid organs [170]. Newer inhibitors have been developed to NSCLC. Nivolumab and pembrolizumab, two PD-1 antagonist antibodies, have been approved by the FDA (Food and Drug Administration) to treat NSCLC patients. Similarly, Atezolizumab, a PD-L1 (programmed death-ligand 1) antagonist antibody, is also FDA approved for NSCLC patients. Ipilimumab, a monoclonal antibody targeting CTLA-4, approved by the FDA in 2011 to treat melanoma patients, is also in phase III trials for NSCLC treatment [167,168,169,170]. Combinatorial antibody and chemo-/radiotherapy approaches are also in phase III clinical trials [171]. In summary, immunotherapy shows considerable promise in the treatment of NSCLC.

Recent studies indicate that autophagy is involved in the innate and adaptive immune response [168]. Autophagy can be stimulated by the innate immune receptors, such as TLRs (Toll-like receptors) and NLRs (nucleotide oligomerization domain (NOD)-like receptors). For example, TLR2 activates JNK and ERK signaling, which in turn stimulate phagocytosis and autophagy, enhancing the host innate immune response [172,173]. In addition, autophagy plays a role in the adaptive immune response, such as antigen presentation and development of lymphocytes (T cells and B cells). Deletion of Atg5 causes not only impaired autophagy, but also lead to defective T cell homeostasis and B cell development [174,175]. Furthermore, autophagy can play both pro-survival and pro-death roles in the context of different immunotherapy compounds [168]. How this occurs is poorly understood, and thus a key area of future study.

### 7.2. Effects of Chinese Medicine on NSCLC

Some traditional Chinese medicines target pathways implicated in cancer progression and survival, including NSCLC, liver cancer, stomach cancer and breast cancer [176]. Several Chinese medicinal compounds cause ER stress induction, which in turn induces autophagy and apoptosis, similar to the actions of curcumin, Licochalcone A and H1 mentioned above. For example, pachymic acid (also called fuling, a lanostane-type triterpenoid from *Poria cocos*) induces G2/M cell cycle arrest in H23 and H460 NSCLC cells by increasing reactive oxygen species (ROS), activating c-Jun N-terminal kinase (JNK) and stimulating ER stress [177]. Furanodiene is a natural terpenoid isolated from Curcumae Rhizoma, which inhibits cell proliferation and induces apoptosis in 95D, A549 and H1299 NSCLC cells via stimulated ER stress evident by increased BiP and CHOP expression [178]. In addition, Platycodin-D, a triterpene saponin extracted from the root of *platycodon grandiflorum*, induces autophagy in both H460 and A549 NSCLC cells exhibited by up-regulation of ATG3, ATG7, Beclin-1 and LC3-II, indicating the autophagic regulation effect of Chinese medicine [179].

## 8. Conclusions and Perspectives

As the most commonly diagnosed and the leading cause of cancer-induced death, non-small-cell lung cancer (NSCLC) remains difficult to cure, with a five-year overall survival rate of around 20% despite various treatment options, including surgical resection, chemotherapy, radiotherapy and targeting therapy. Determining the molecular mechanisms of NSCLC and the discovery of new biomarkers will aid in the development of better, more specific therapeutics.

Several gene aberrations are associated with NSCLC, promoting oncogenesis and disturbing cellular homeostasis. These genes affect not only their own functions, but have a more widespread effect due to their abnormal activity, feedback loops and crosstalk between different signaling pathways, meaning that single agents may be insufficient to effectively inhibit tumor growth. Instead, co-treatments may achieve better curative effects in the clinic.

Apoptosis and autophagy are two important physiological activities that control cell survival and cell death. The crosstalk between autophagy and apoptosis influences cell homeostasis, cargo clearance of dying cells, as well as clinical therapeutics. In this review, we summarized the most investigated factors and signaling pathways associated with autophagy and apoptosis in NSCLC, including abnormal genomes, aberrant mTOR pathway and ER stress. Further uncovering the precise nature of this elaborate and highly complicated crosstalk will have broad pathophysiological implications.

The relationship between autophagy and apoptosis is controversial and highly dependent on the context and the specific molecules that are used for treatment. In some circumstances, autophagy counteracts apoptosis, whereas in other situations, autophagy acts synergistically with apoptosis. Appropriate application of stress could theoretically tip the balance in favor of more effective cancer therapeutics, wherein transient and low doses of stress could induce autophagy, inhibit apoptosis and mediate cellular homeostasis, whereas persistent and high doses of stress might induce apoptosis. Alternatively, switch proteins or complexes may act as “guardians” that evaluate the cellular context and ultimately define which role autophagy will play during apoptosis. The apoptotic effect elicited by autophagic proteins, as well as the autophagic effect by apoptotic proteins has been discussed, highlighting the crosstalk between both processes. The use of various methods and techniques, such as co-immunoprecipitations and mass spectrometry, will help further our understanding on the crosstalk between autophagy and apoptosis and help further our understanding on how best to target these processes to treat cancers like NSCLC.

## Figures and Tables

**Table 1:** Genetic abnormalities in non-small-cell lung cancer (NSCLC).

Genetic Alterations	Incidence (%)	Incidence (%)
Mutation	Adenocarcinomas	Squamous-Cell Carcinoma
*p53*	45–70	60–80
*KRAS*	10–30	
*EGFR*	10–40	
*LKB1*	34	19
*MET*	14	
*DDR2*		4
*HER2*	4	
*BRAF*	2–10	3
*PTEN*	2–5	3–10
*PIK3CA*	2	2–18
*AKT1*		2
**Fusion**		
*ALK*	7	
*ROS1*	1–2	
*RET*	1–2	
**Amplification**		
*MET*	5–20	3–21
*TITF-1*	15	15
*EGFR*	15	30
*HER2*	6	2
*PIK3CA*	6	25–45
*FGFR1*		16–25
**Deletion**		
*CDKN2A*		51
*PTEN*	20–40	20–60

## Abbreviations

NSCLC	Non-small-cell lung cancer
SCLC	Small-cell lung cancer
mTOR	mammalian target of rapamycin
mTORC1	mTOR complex 1
mTORC2	mTOR complex 2
BID	BH3 interacting-domain death agonist
Bik	Bcl-2-interacting killer
Noxa	Phorbol-12-myristate-13-acetate-induced protein 1
HRK	Activator of apoptosis harakiri
PUMA	BCL2 Binding Component 3
Bax	BCL2 Associated X
Bak	Bcl-2 homologous antagonist/killer
Bim	BCL2-Like 11
Bcl-2	B-cell lymphoma 2
Bfl-1	BCL2 Related Protein A1
MCL-1	Myeloid Cell Leukemia Sequence 1
Fas	Tumor Necrosis Factor Receptor Superfamily, Member 6
RTK	Receptor tyrosine kinase
PI3K	Phosphatidylinositol-3 kinase
TNFα	Tumor necrosis factor alpha
TNFR1	TNFα Receptor 1
TRADD	TNFR1-associated death domain
TRAIL	TNF-related apoptosis inducing ligand
FADD	Fas-associated protein with death domain
NF-κB	Nuclear factor kappa-light-chain-enhancer of activated B cells
MDM2	Mouse double minute 2 homolog
MOMP	Mitochondrial outer membrane permeabilization
ER stress	Endoplasmic reticulum stress
DISC	Death-inducing signaling complex
FIP200	FAK family kinase-interacting protein of 200 kDa
ULK1	UNC-51-like kinase 1
IGF-1	Insulin-like growth factor-1
HER	Human epidermal growth factor receptor
VEGFRs	Vascular endothelial growth factor receptors
IRS	Insulin receptor substrate
PtdIns(4,5)P_2_	Phosphatidylinositol 4,5-bisphosphate
PtdIns(3,4,5)P_3_	Phosphatidylinositol (3,4,5)-trisphosphate
PtdIns(3,4)P_2_	Phosphatidylinositol 3,4-bisphosphate
PTEN	Phosphatase and tensin homolog
PDK1	3-Phosphoinositide-dependent protein kinase-1
PP2A	Protein phosphatase 2
LKB1	Liver kinase B1
ERAD	Endoplasmic-reticulum-associated protein degradation
